# Harnessing Social Media to Enhance Nephrology Academia

**DOI:** 10.31729/jnma.8268

**Published:** 2023-09-30

**Authors:** Priti Meena, Subashri Mohanasundaram, Jithu Kurian, Ganesh Srinivasa Prasad, Vinant Bhargava, Sandip Panda, Krishna Kumar Agrawaal

**Affiliations:** 1 Department of Nephrology, All India Institute of Medical Sciences, Patrapada, Bhubaneswar, India; 2 Department of Nephrology, Government Medical College & Hospital, Thoothukudi, Tamil Nadu, India; 3 Pushpagiri Medical College Hospital, Thiruvalla, Kerala, India; 4 Mazumdar Shaw Medical Center, Narayana Health, Bengaluru, India; 5 Department of Nephrology, Sir Ganga Ram Hospital, Rajinder Nagar, New Delhi, India; 6 Department of Internal Medicine, Universal College of Medical Sciences, Bhairahawa, Rupandehi, Nepal

**Keywords:** *education*, *nephrology*, *social media*

## Abstract

The process of learning has been confined to the realms of educational institutions. Over the last ten years, the semantics of social media networks have evolved with the use of mobile gadgets. Consequently, nephrologists have realised the potential benefits of using these platforms for their educational and career development. Social media can change the horizon of nephrology education. The concept of bedside examination, teaching and sharing experiences have changed with the advent of Facebook, YouTube, Instagram and X (former Twitter). Other networking portals, such as WhatsApp, Telegram, X (former Twitter), and Pinterest, have also amassed the attention of selected users. Despite split opinions on the utility of social media, it is undeniable that it has influenced interaction between students and mentors. Resources ranging from online networks, blogs, visual aids, podcasts, online journal clubs, videos, live conference coverages, and tutorials have made it possible for nephrologists to stay informed and educated with recent updates. In this review, we discuss how social media can enrich nephrology academia, facilitate the sharing of research and access to fellowships and mentorship programs, provide career prospects to trainees, and broadcast scientific conferences while bringing nephrology societies together.

## INTRODUCTION

With cutting-edge advances in the medical field, innovative modalities for the dissemination of knowledge have become a need for the masses. In the last decade, medical technology has been advancing at an unprecedented rate, and social media has become the go-to solution for providing easily accessible and up-to-date medical literature, as well as for keeping abreast of the latest training methods.^1,2^ In this regard, Social media in nephrology offers a plethora of options, ranging from providing information about fellowship training, the latest research articles, conferences updates and employment opportunities to simplifying and explaining complicated nephrology topics in the form of tweetorials, blogs, infographics, online journal clubs, podcasts, videos.^3,4^ In this review article, we have focused on the scope of social media in improving nephrology education, promoting free idea exchange, and disseminating updated research within a matter of a few clicks.

## TRADITIONAL VERSUS ONLINE EDUCATION

In the early days of education, students used to stay with their teachers to complete their formal education through schools, colleges, and universities.^5^ Currently, information technology has made education possible from the comforts of our homes from a teacher far end of the planet. The method of traditional education has evolved from classical didactic lectures to group discussions and activities to encourage student participation.^6,7^

Online learning is one of the economical forms of education that allows students to learn even in places with limited resources and minimal equipment by using audio-visuals, adaptive tutorials, virtual models, etc., for theoretical teaching as well as for practical demonstrations.^8^ Using this platform, medical students also have the opportunity to interact and learn directly from experts in their field through online video lectures, apps, courses, webinars, conferences, etc. from all around the world. A wealth of information is available online, which is continuously updated according to the most up-to-date guidelines and trends in the medical field. Additionally, this platform makes use of various teaching aids and digital tools. Incorporation of teaching aids, such as video tutorials, case studies, virtual patients, animations, and e-projects, in online learning, improves learning by engaging students and prevents stagnancy. The COVID-19 pandemic has been an impetus for the increased use of online learning in college education, which facilitates convenience and access to information regardless of physical location.^9^

## INTRODUCTION TO SOCIAL MEDIA

As of July 2022, 59% of the world's population which is approximately 4.7 billion people, use social media.^10^ Usage has risen exponentially in the past decade, growing at an annual rate of 5.1% and with more than 7 new users every second. Popular platforms such as Facebook (2.936 billion monthly active users), YouTube (potential reach of 2.476 billion), WhatsApp, Instagram, Telegram, Twitter, Pinterest and LinkedIn can reach out to a large population.^11^ Notably, the frequency and intensity of usage are less in countries with low to middle incomes. Retrospective analysis has shown that the availability and accessibility of the internet are somehow related to the country's gross national income (GNI) per capita.^12^

## SOME TOOLS IN NEPHROLOGY EDUCATION

**Figure 1:**
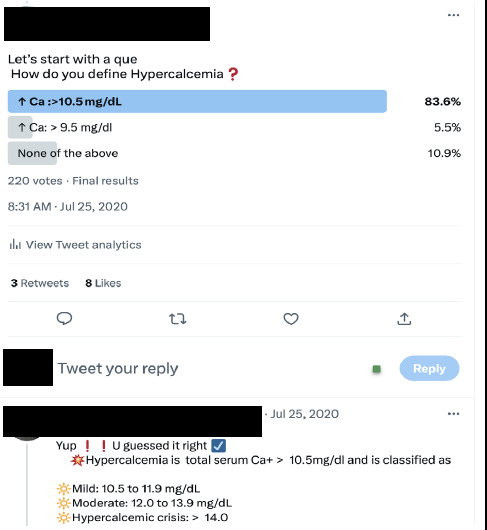
Example of a tweetorial.

Microblogging: Microblogging is posting brief messages or fragments of texts, pictures, short videos or audio clips on social media. These fragments of content are referred to as Tweets, on the X platform.^13^ The bright side of these tweets is that it has taken social media from being a platform for announcing one's achievements and success stories to creating a conducive environment for letting people know about scholarships, fellowship programs, and links to the latest guidelines, all in a very concise format. Microblogging's short-form communication format encourages engagement by allowing users to like, share, comment and retweet. For example, a tweet is a piece of content posted by a user whose identity is indicated by their handle, which is represented by a @ sign and a string of letters or numbers. Handles can stand in for hospitals, academic journals, medical schools or residency programmes, or even specific people. The maximum character count for a tweet is 280, however, X allows many tweets to be chained together as a thread. Tweetorials are organized in an interactive way with polls asking questions, progressively revealing diagnostic hints, and allowing for questions and responses.^14^ Tweets frequently include hashtags, allowing for the indexing of content according to words or phrases that serve to connect the tweet to related content (Figure 1).

An effective way of microblogging for educational purposes is to share articles or videos along with the initial tweet, as this invites more audiences to interact with the content. Also, creating a thread of shorter posts, with illustrations, makes reading the content easier.

**Blogs:** Blogs are succinct posts usually composed in plain language and posted on sites to give knowledge about a particular topic.^15^ Since numerous blogs incorporate dynamic comment segments, these posts regularly fill in as a gathering place for conversations. Intriguing and instructive blog entries in Nephrology are NephJC, NephSIM, Nephron Power, Precious Bodily Fluids, Renal Fellow Network, Last Month in Nephrology, GlomCon, and AJKD blogs.^16-21^ Another nephrology blog region that is endorsed by AcademicCME is the KI Reports Community (which also includes Little Beans). It offers a wide range of data, including tweetorials, article summaries, and infographics.^22^

**Audio:** Podcasts, digital audio files that are to be played on phones or MP3 players, have become a popular form of medical teaching. The development of medical podcasting has advanced during the last 10 years as producers explore and change format.^23^ Additionally, the production of podcasts has moved from being a component of conventional medical learning locations such as journals and medical school courses to natural digital learning, made by individuals outside or in conjunction with the traditional medical education system. Some informative podcasts in nephrology are Freely Filtered-A NephJC Podcast, Global Kidney Care Podcast Provided by ISN, Channel Your Enthusiasm The Nephron Segment: A Kidney Podcast, CJASN Podcast and ASN Podcast.^24-26^

Another audio aid is X Spaces which is a feature in the X app that allows users to create and join audio chat rooms.^27^ It is similar to a podcast, but takes place in real-time and is open to anyone. On Spaces, people can share their ideas and knowledge, just like they would on Twitter, but in a live audio format. Spaces are unedited and open to anyone, making them inclusive and accessible. To sum it up, X Spaces is a great place for people to have conversations and exchange ideas with others who hold similar views. The International Society of Nephrology, for instance, often uses Spaces to discuss relevant topics in nephrology, including patient perspectives. Recordings of these spaces by ISN are also available on the site.^28^

**Use of Messaging apps:** One of the most widely used messaging apps is WhatsApp. It is a free and simple-to-use program that enables users to exchange images, videos, audio recordings and text messages.^29^ This app can be used to share information, facilitate conversations and even conduct virtual classes. Nephrologists are taking advantage of this by making use of WhatsApp groups to post new articles, exchange information, discuss intriguing cases, and challenging interventions and plan meetings. Similarly, the use of the Telegram app in education is becoming increasingly popular. Slack is another platform for users to communicate effectively in real time, share files and resources, work together on projects, make announcements and discuss various topics.^30^ It especially excels at quick and media-rich interactions. The service also provides threaded messages, which are a series of comments based on an initial message. Private talks between two individuals and chat rooms for a smaller group of collaborators are also available. Furthermore, Slack can be integrated with other programs such as Zoom, Google/Outlook Calendar, Airtable, Simple Poll, and many others. Several nephrology committees including, the Nephrology Social Media Collective internship (NSMC) and ISN Education team are taking advantage of Slack to enable members from around the world to converse and complete tasks in real-time.^31,32^

**Visual Abstracts:** Infographics and visual abstracts are great ways to capture a reader's attention and spread the results of research. Visual abstracts are useful because they make complex information easier to understand quickly. Recently, they have become very popular on social media as a way to promote research. They provide a short synopsis of data on paper, usually in the form of graphics, icons, charts, or illustrations with minimal accompanying text, by which, readers can decide if they want to delve into the full article or not.^33^ Several nephrology journals such as the American Journal of Kidney Diseases, Clinical Journal of the American Society of Nephrology (CJASN), Kidney International, Kidney 360, Journal of the American Society of Nephrology, Nephrology, Dialysis and Transplantation (NDT) and Indian Journal of Nephrology have now adopted the use of visual abstract alongside the publication of important studies. Most nephrology journals have a dedicated team to make visual abstracts which are then reviewed by authors to finalize them while some journals ask respective authors to provide visual abstracts for their manuscripts. These journals usually provide appointed pre-decided templates to create visual abstracts.^34^ Software such as Powerpoint, Inkscape, Canva, Adobe Illustrator, bio-render and Corel Draw can be used to create visual abstracts which can then be saved and shared in image form. Generally, a visual abstract consists of details about the cohort and patients, methodology, key findings and conclusion of a study. Previous studies have revealed that visual abstracts can enhance visibility and the number of clicks on the article link, however, no increase in full-text views of the article was found.^35,36^ Nevertheless, there are some disadvantages such as oversimplification, risk of misinterpretation and chances of inaccuracy and misrepresentation of data.^36^

**Nephrology journal updates:** Major journals in the field of nephrology, such as Nature Reviews in Nephrology, NDT, The New England Journal of Medicine, Nephron, Clinical Kidney Journal, Kidney Medicine, American Journal of Kidney Diseases, Indian journal of transplantation, American Journal of Nephrology, CJASN, Kidney 360, Journal of the American Society of Nephrology, and Kidney International, have begun to share their new articles through social media sites like Twitter, Facebook, and Instagram.^4,26,37^ This allows for the possibility of open dialogue and interaction with readers. Additionally, the purpose of this effort by the journals is to increase their readership; however, it is not yet known if this will lead to an increase in the number of citations of any given article. Readers can follow the accounts of these journals on social media and receive notifications whenever new content is released.

**Conference coverage:** Various nephrology conferences happen at regular intervals which help us learn, relearn and update our knowledge. However, it is nearly impossible for many to attend every CME due to various reasons including, shortage of time, travel, and cost. Organizers of almost all conferences are now forming dedicated social media teams to cover conferences, where people in these social media teams give live updates. These X can be seen with the trending hashtags of the conference.^38^

**Figure 2:**
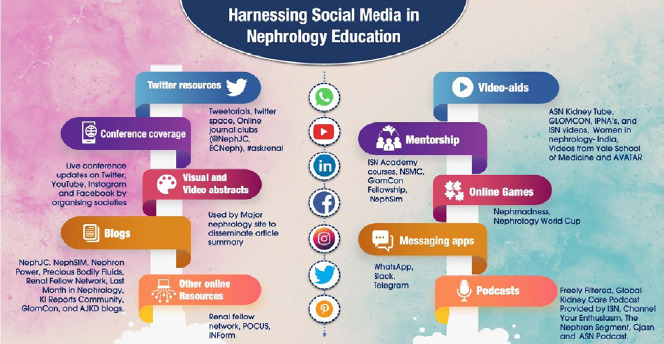
Tools on social media for nephrology education.

**Videos:** YouTube is one of the most popular eminent sources of e-knowledge on the web for both medical professionals and patients. It allows them to collaborate by sharing, editing, and discussing educational content. Videos are especially helpful for learning in an online or blended environment, as they are typically the focal point of these courses.^39^ Students often find videos to be more engaging due to their convenience, succinctness, and the fact that they can be watched in various settings to supplement their clinical knowledge. It is also possible to comment on videos, share files, ask questions and answer those of others. There are plenty of videos that cover topics such as renal physiology, procedures for renal biopsy and hemodialysis catheter, lectures and conference sessions, with some of the noteworthy channels for nephrologists being the Yale School of Medicine, Women in Nephrology-India, ASN Kidney Tube, GlomCon, IPNA's video library and the International Society of Nephrology videos.^4,26,37,39^"^41^ ISN organizes Nuances in Nephrology live webinars on nephrology topics where participants can interact with the specialist presenter and clear up any questions.^41^ Various tools are shown below (Figure 2).

**Interactive learning:** Social media help us to connect with vast numbers of people across the world and we can learn about real-world experiences of various disease management. On various social media platforms such as X, Mastodon, and Reddit everyone can share, discuss and learn together. The #AskRenal tool is a popular interactive learning system on Twitter, developed by social media nephrologists.^25^ Nephmadness is a non-profit, learning program that gives Continuing Medical Education credits and uses evidence-based practices.^43^

**Online Journal Clubs:** Twitter-based journal clubs (hashtag #twitjc, @TwitJournalClub) have emerged, bringing together doctors and students from all around the world. The way health professionals interact with one another for lifelong learning is changing as a result of these online interfaces. Nephrology Journal Club (NephJC) is an important example of an interactive online journal club.^44^ It is centred on Twitter, and a summary of the latest landmark nephrology article is released on the NephJC website. Authors or invited specialists frequently participate to offer clarification and to respond to queries. Other interactive Twitter-based nephrology journal clubs are #IPNAJJC, pediatric nephrology #UroJC, urology #GenMedJC, internal medicine #PathJC, pathology, #HYPHIP (hypertension), #IWINChat Women in Nephrology-India tweet chats. #ECneph (Everyday Cases in Nephrology) is a bi-monthly case-based education program that takes place on Twitter using the hashtag #ECneph.^45^ A moderator will present unique or challenging cases from their nephrology clinics or wards which are then discussed by nephrologists from around the world for one hour on thursday nights.

**Mentor-Mentee Programs:** An essential component of medical education is the mentor-mentee relationship. social media opened a global platform, where early career nephrologists are given crucial professional advice by mentors, while mentees bring variety and new ideas to labs and clinics. The whole purpose of a mentorship relationship is goal-setting. Mentors are expected to create a time frame and a specific action plan to aid their proteges in achieving their dreams. The NSMC internship intends to develop leaders in the use of social media in medicine by imparting knowledge, skills, attitudes, professionalism, and competence.^46^ NephSim and GlonCom are other overseas online programs where one can look for mentorship from expert nephrologists.^47,48^

**Online fellowships:** Nephrology trainees are regularly taking advantage of the increased information and access to online fellowships in nephrology and creating their career paths. The ISN Academy provides multiple online courses, instructed by experts in the field.^49,50^ GlomCon's Virtual Glomerular Disease Fellowship is created to help trainees develop their understanding of the diagnosis and treatment of people who have glomerular diseases.^48^

**Other online resources:** Point of Care of Ultrasonography (POCUS) is another resourceful website that holds a large selection of ultrasound images, cases, infographics, and visual abstracts.^51^ The Interventional Nephrology Forum (INForm) is an excellent source of information, with video recordings of sessions organized by the INForm group available.

**Figure 3:**
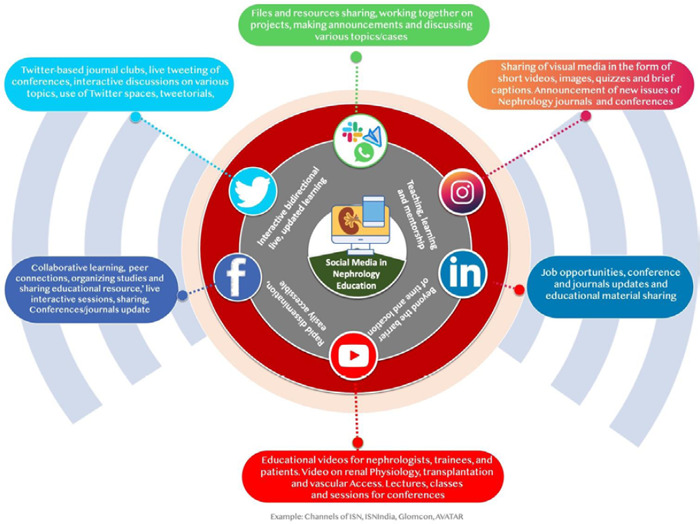
Social media platforms in nephrology education.

These videos and data are specifically related to cases, procedures and issues encountered when working in interventional nephrology (Figure 3).^52^

## WAY FORWARD

The potential benefits of using social media as a platform for nephrology education with the latest advances could be undermined by the spread of false information.^2^ Materials that are posted on social media, are usually not reviewed by experts and can be, therefore, misleading and may lead to sharing of or commercially driven data. Evidence-based medicine usually does not rely on personal accounts or experiences. Yet social media often does so, using individual patient experiences to build collective medical knowledge.^53^ Furthermore, trolling can be a problem, disrupting conversations and leading to the spread of inaccurate information regarding treatments and outcomes of chronic diseases. When sharing educational content, such as medical images or case histories, it is essential to gain patient consent and keep data confidential, as patients may not be willing to share their information. Engaging with social media comes with potential drawbacks, such as the possibility of posting content that could create a negative perception of healthcare professionals, students, and associated educational facilities. Furthermore, practitioners may act inappropriately if they utilise data found on social media without a complete understanding of the shared information in an inappropriate manner to treat their patients. The future holds great promises for social media, as it is emerging to be a powerful tool for disseminating the latest knowledge, improving education standards with updated training modules, and connecting masses across the globe to share experiences and improve outcomes in health care delivery.
